# Associations between domestic violence and psychosomatic symptoms and health behaviors during pregnancy and the puerperium: a comparative cross-sectional study

**DOI:** 10.3389/fgwh.2025.1557543

**Published:** 2026-01-12

**Authors:** Ana Bertha Zavalza-Gómez, Andrea Meza-Martínez, Sergio Jiram Vázquez-Sánchez, Evelin Castillo-Martínez, Samantha Emily González-Muñoz, Andrea García, Kathia Dayana Morfín Meza, Alejandro González-Ojeda, Gabino Cervantes-Guevara, Enrique Cervantes-Pérez, Sol Ramírez-Ochoa, Andrea Socorro Álvarez-Villaseñor, Ana Olivia Cortés-Flores, Guadalupe Castillo Cardiel, Clotilde Fuentes-Orozco, Ramirez Velazquez Alejandro

**Affiliations:** 1 Research Department, Gynecology and Obstetrics High Specialty Medical Unit of the Centro Médico Nacional de Occidente del Instituto Mexicano del Seguro Social, Guadalajara, Jalisco, Mexico; 2 Biomedical Research Unit 02, National Medical Center of the West, Mexican Social Security Institute, Guadalajara, Jalisco, Mexico; 3 Faculty of Medicine of the Universidad de Colima, Colima, Colima, Mexico; 4 Department of Wellbeing and Sustainable Development, Centro Universitario del Norte, University of Guadalajara, Colotlán, Jalisco, Mexico; 5 Department of Internal Medicine, Hospital Civil de Guadalajara Fray Antonio Alcalde, Guadalajara, Jalisco, Mexico; 6 Assistant Medical Coordination for Health Research, Mexican Social Security Institute, La Paz, Baja California Sur, México; 7 Department of Oncologic Surgery, ONKIMIA, Guadalajara, Jalisco, México; 8 Department of Maxillofacial and Plastic and Reconstructive Surgery, Specialties Hospital, Western Medical Center, Mexican Institute of Social Security, Guadalajara, Jalisco, Mexico

**Keywords:** domestic violence, postpartum period, pregnancy, preterm birth, psychosomatic

## Abstract

**Background:**

According to the WHO, violence is the intentional use of force or power against oneself, another person, or a community, causing injury, death, or harm. National data from 2021 reveal that as many as 70.1% of women in Mexico have experienced some form of violence. Pregnancy represents a period of heightened vulnerability, with negative impacts on material and infant health.

**Material and methods:**

This cross-sectional observational study was conducted on women during the immediate postpartum period. Domestic violence was assessed using the Woman Abuse Screening Tool (WAST) scale, the Affective Bonding and Prenatal Adjustment Assessment Scale survey, and postpartum depression was evaluated through the *Centre for Epidemiological Studies-Depression Scale (*CES-D) scale.

**Results:**

Four hundred women were included using the short version of the WAST scale, with 31.5% (126) testing positive for the screening questions. Women who experienced abuse showed higher odds of postpartum depression (OR = 2.95; 95% IC: 1.87–4.63; *p* = 0.000) and tobacco use (OR = 2.28; 95% IC: 1.31–3.94; *p* = 0.003). Adverse perinatal effects, such as preterm delivery (35.7%), admission to intensive care (40.5%), and low birth weight (19.8%), were frequent but without statistical significance.

**Conclusions:**

One-third of the pregnant women in the study suffered intimate partner violence, mainly psychological and economic. Unwanted pregnancy, lack of emotional bonding, postpartum depression, alcohol/smoking, and low education levels were frequent factors.

## Introduction

Domestic violence (DV) is defined as a pattern of abusive behavior used by one partner to gain or maintain power and control over another. It may involve physical, sexual, emotional, economic, psychological, or technological actions or threats, as well as other coercive behaviors intended to intimidate, manipulate, humiliate, isolate, frighten, terrorize, coerce, threaten, blame, hurt, injure, or wound someone (1).

The World Health Organization (WHO) reports that violence perpetrated by an intimate partner constitutes the most common form of physical and sexual violence experienced by women worldwide (2). The global prevalence of this form of violence varies widely by country, from 6% to 68% (3). Overall, approximately 30% of women have been victims of either physical or sexual violence by a partner, or sexual violence committed by someone other than their partner. Among women aged 15–49 who have been in a relationship, 27% report that have been subjected to some form of physical and/or sexual violence from an intimate partner (1).

In Mexico, the prevalence of DV against women reached 11.5% in 2021, decreasing mid-year before rising to 16.3% in December. In that same period, national data indicated that 70.1% of women aged 15 years or older had experienced at least one form of violence (psychological, economic, patrimonial, physical, sexual, or discriminatory) at some point in their lives. Psychological violence was most common, followed by sexual, physical, and economic or discriminatory forms (4, 5).

In this matter, health consequences of DV in women are not limited to physical injuries such as fractures, contusions, lacerations, and internal organ damage (6). DV also has a substantial impact on mental health, with exposed women receiving significantly more diagnoses of generalized anxiety, dysthymia, depression, phobias, harmful alcohol consumption, and psychoactive drug dependence than those who report no abuse, ultimately contributing to declines in overall functioning (7). Violence against women, especially during pregnancy, constitutes a serious public health problem (8). This type of violence, whether physical, sexual, or psychological, requires priority attention from health services due to the significant risks to maternal and child health. Women who are victims of intimate partner abuse are 16% more likely to suffer a miscarriage and 41% more likely to have a pre-term birth, and almost twice as likely to suffer from depression (1).

Different studies show that the experience of violence during pregnancy can cause problems such as obstetric issues, premature rupture of membranes, urinary tract infections, vaginal bleeding, premature weaning from breastfeeding, and prenatal depression (9–11). It also increases the possibility of perinatal and neonatal mortality (12). In this sense, motherhood is experienced more satisfactorily with social support, a healthy couple relationship, a positive emotional disposition, and reduced exposure to stressors, as these conditions facilitate adaptation, the development of bonding with the child, and compatibility with the maternal role. Therefore, assessing prenatal bonding and adaptation is essential to identify negative attitudes or behaviors and implement timely interventions, especially the potencial harm to the fetus through inadequate prenatal care, alcohol consumption, or physical violence. To support such evaluations, various instruments have been developed to assess these prenatal processes (13–17).

Increasing evidence, along with organizational support, has promoted the adoption of systematic screening programs aimed at identifying women who are experiencing abuse. Within non-specific surveillance surveys of intimate partner violence (IPV) against women, the Woman Abuse Screening Tool (WAST) was developed to detect emotional or physical abuse by a partner (18). A recent investigation affirmed the validity of the WAST in terms of its internal structure, measurement invariance, convergent validity, clinical validity, and reliability, thereby supporting its application for detecting potential cases of IPV and enabling timely intervention (19).

Although DV is highly prevalent in Mexico and has serious implications during pregnancy, studies addressing this issue in the Mexican population are scarce. Updating the evidence is crucial to understand the current impact of DV on maternal health and to guide effective preventive and clinical strategies.

Our study aims to analyze the relationship between exposure to DV and the development of psychosomatic symptoms and health behaviors during pregnancy and the puerperium in Mexican women, focusing on DV screening, its impact on parent-fetal bonding, and the comparison of maternal and neonatal outcomes between exposed and unexposed patients.

## Material and methods

### Study design

An analytical observational cross-sectional study was conducted. Patients were included from July 1, 2022, to February 1, 2024, of female sex of any age, who were during the immediate puerperium period, after instrumented uterine curettage, and after manual vacuum aspiration, who were being evaluated at the High Specialty Medical Unit, Obstetrics and Gynecology Hospital of the Centro Médico Nacional de Occidente.

#### Patient selection

A total of 400 women of any age were included in the study. All participants were evaluated during the puerperal period following vaginal delivery, cesarean section, instrumented uterine curettage, or manual vacuum aspiration. Exclusion criteria included being under sedation, admission to the obstetric intensive care unit, tracheal intubation, a clinical diagnosis of depression or psychosis under active treatment, and incomplete questionnaires.

#### Sample size

A non-probability convenience sampling method was used, including all participants who met the inclusion criteria and agreed to take part in the study.

A minimum sample size of 323 women was determined using the formula for proportions, based on a reported prevalence of domestic violence of 30% as cited by the WHO (1), with an anticipated margin of error of 5%, a confidence level of 95%, and a statistical power of 80%.

The parameters applied in the calculation were: *zα* = 1.96 (confidence level), *p* = 0.30 (probability of the event), *q* = 0.70 (probability of non-event), and *δ* = 0.05 (precision). The sample size calculation was as follows:

$$n=(1.96{)}^{2}\times (0.30)\times (0.70)/(0.05{)}^{2}=(3.84)\times (0.21)/(0.0025)=323$$

To account for an estimated 20% non-response rate, the final sample size was adjusted to include at least 387 women (20).

### Variables

Independent variable: Domestic violence experience.

Dependent variables: Psychosomatic symptoms, negative health behaviors during pregnancy and the Puerperium (alcohol abuse, smoking, drug abuse, sleep issues).

Confunders: Age, socioeconomic status, parity, education/occupation.

Groups: Based on the Women Abuse Screening Tool (WAST), the women were categorized into two groups: a positive group, defined by meeting criterion 1 (score 1-1-0) or criterion 2 (score 1-0-0), which indicates a possible case of intimate partner violence, and a negative group, characterized by a score of 0-0-0 on all three WAST items.

#### Data collection

Between July 1, 2022, and February 1, 2024, participants were recruited from the obstetric hospitalization area of the High Specialty Medical Unit, Obstetrics and Gynecology Hospital at the Centro Médico Nacional de Occidente. Women who agreed to participate provided written informed consent and were subsequently interviewed regarding sociodemographic characteristics (age, education, occupation, marital status, place of origin, religion, duration of cohabitation with their partner), gynecologic and obstetric history (parity, history of abortions, gestational losses, cesarean sections, desired and/or planned pregnancy, hypertensive disorders, glucose levels, thyroid function, obstetric hemorrhage, pathological personal history, the threat of abortion, need for adult intensive care unit admission, preterm delivery [gestational age], low birth weight, intrauterine growth restriction, perinatal death, immunological or infectious diseases in previous or current pregnancies), and negative health behaviours, including substance and alcohol use, smoking, and delayed prenatal care. Information on severe outcomes such as homicide or suicide risk was also collected. After the interview, participants completed the short version of the Women Abuse Screening Tool (WAST), which has been translated into Spanish and validated in Spanish-speaking populations (21, 22). Administration of the interview and the screening instrument required approximately 40 min.

The Evaluation of Affective Bonding and Prenatal Adaptation (EVAP) scale was then administered, requiring approximately 15–20 min, followed by the Centre for Epidemiological Studies-Depressión Scale (CES-D), which required a similar duration. Additionally, medical records were reviewed to corroborate maternal and neonatal diagnoses documented by healthcare personal. Maternal weight and height were retrieved to calculate body mass index (BMI) and classify participants as underweight, normal weight, overweight, or obese. For newborns, data on weight, length, Apgar scores, gestational age assessment (Capurro or Barrard), birth characteristics, and maternal-fetal complications recorded in the clinical file.

The instruments were administered by two researchers, who were previously trained for both the application of the surveys and psychiatric care, if necessary, during the interrogation, to avoid probable bias. All interviews were conducted privately, free of interruptions inside the hospital, when the women went to social work and nursing for pre-discharge care, counseling, and postpartum family planning. Each month, the research team checked the quality of the records and collected the information.

#### Measurement instruments

##### Woman abuse screening tool (WAST)

Originally developed in English for use in primary care, this test consists of seven items designed to identify emotional or physical partner abuse. The first two questions assess the level of tension and difficulty within the relationships, while the remaining items explore the frequency of emotional, physical, and sexual violence (18). Notably, the short version of the WAST, which includes only these initial two questions, has demonstrated acceptable validity indices for screening IPV in the general population, thereby reinforcing its utility as a concise yet effective assessment tool (22, 23).

##### Affective bonding and prenatal adjustment assessment scale (EVAP)

This scale generally assesses two main dimensions (affective bonding and prenatal adaptation) through 30 multiple-choice items. The affective bonding dimension examines the mother's emotional connection with the fetus through 3 components: cognitive engagement, the ability to perceive and differentiate the fetus as a separate being, and the quality of her interactions with it. The prenatal adaptation dimension examines acceptance of pregnancy, adjustment to motherhood, childhood experiences, prenatal care, and emotional well-being (24). In 2019, a study provided validity evidence and introduced a shortened 21-item version encompassing the same two dimensions. Reliability findings indicated satisfactory coefficients for all (25).

##### Centre for epidemiological studies-depression scale (CES-D)

The CES-D scale is a self-reported psychometric instrument designed to identify the frequency and severity of depressive symptoms. It consists of 20 items measured on a four-point Likert scale and is organized into four underlying factors: depressed affect, positive affect, somatic complaints, and interpersonal difficulties (26). Recent research has demonstrated that the CES-D possesses high diagnostic accuracy and can be recommended for use as a first-stage depression screening method in adults (27).

### Statistical analysis

The Statistical Package for the Social Sciences v. 23 program was used for data analysis and the database was created in Excel. Descriptive statistics were used for data analysis; continuous variables are given as mean and standard deviation and categorical variables as frequencies and proportions. Categorical variables were compared using chi-square test, and results were reported as odds ration with 95% confidence intervals. The relationship between violence and the variables studied in the woman and the aggressor was determined employing a multiple linear regression model with response variable, the total score of the WAST instrument, and demographic variables: age, schooling, marital status, occupation, number of children, history of violence in the family of origin, partner support and family support; and explanatory variables of the partners: age, schooling, occupation, alcohol consumption, and drug consumption. A value of *p* ≤ 0.05 was considered statistically significant.

### Ethical approval

The study was conducted in accordance with the ethical principles outlined in the Declaration of Helsinki and its subsequent amendments, the General Health Law, and all relevant institutional regulations governing research involving human subjects. Ethical approval was granted by the Local Health Research and Ethics Committee of the Hospital de Gineco-obstetricia, Centro Médico Nacional de Occidente, Guadalajara, Jalisco, Mexico, on June 21, 2022, under registration number R-2022-1310-057. Informed consent was obtained from all participants.

Confidentiality and anonymity were rigorously upheld throughout the research process. Participants were identified exclusively by initials and a sequential numerical code within the study database to ensure the protection of personal information. Access to clinical records was restricted to the principal investigators and was managed in accordance with applicable legal and institutional regulations. All data collected were used solely for research purposes.

## Results

A total of four hundred postpartum women were interviewed. The sample consisted predominantly of young adult women, with a mean average of 29 years, and most participants reported their sexual debut during late adolescence. The majority had experienced two pregnancies on average. Anthropometric measures indicated elevated body weight in the population, with mean values consistent with overweight status (Table 1).

**Table 1:** Sociodemographic characteristics of the study population.

Variable	Mean ± SD	Mínimum	Máximum
Age (years)	29 ± 6.23	13	44
Age at sexual debut (years)	18 ± 6.19	13	36
Number of pregnancies (gravidity)	2 ± 1.33	1	7
Weight (kg)	78 ± 17.12	48	143
Height (cm)	160 ± 6.43	144	183

In terms of sociodemographic characteristics, most participants had completed middle to high levels of formal education, with a smaller proportion holding higher education degrees, and almost none reported no schooling. Marriage and cohabitation were the predominant marital arrangements, whereas single or divorced women represented a minority. Employment was evenly distributed, with similar proportions of employed and unemployed participants. According to BMI categorization, excess weight was highly prevalent, with overweight and obesity representing the majority of the sample, while normal weight was observed in a minority of participants (Table 2).

**Table 2:** Sociodemographic characteristics of the study population. (Continuation).

Variable	Frequency	Percentage
Educational level		
None	1	0.3%
Elementary school	24	6.0%
Junior High	142	35.5%
High School	129	32.3%
Bacheloŕs degree	98	24.5%
Master's degree/PhD	6	1.5%
Marital status		
Single	46	11.5%
Cohabiting	175	43.8%
Married	176	44.0%
Divorced	3	0.8%
Employment status		
Unemployed	201	50.2%
Employed	199	49.7%
Nutritional status according to BMI		
Normal weight	59	14.7%
Overweight	126	34%
Obesity grade I	115	28.7%
Obesity grade II	53	13.2%
Obesity grade III	46	11.5%
Obesity grade IV	1	0.2%

Using the short version of the WAST scale, participants were screened for domestic violence. A total of 126 women (31.5%) screened positive, whereas 274 (68.5%) screened negative. Of the 126 women who tested positive for domestic violence, 108 (27.0%) reported some tension in response to the question, “*How would you describe your relationship with your partner?*” In response to the second question, “*You and your partner resolve your arguments with:*”, 130 (32.5%) indicated some difficulty. Additionally, according to the interpretation criteria, 107 (26.8%) were positive for criterion 1, and 15 (3.8%) were positive for both criteria 1 and 2 (Table 3).

**Table 3:** Results screening questions of WAST scale for domestic violence.

Question 1: In general, how would you describe your relationship with your partner?		
	*N*	%
A lot of tension	18	4.5
Some tension	108	27
No tension	274	68.5
Question 2: You and your partner resolve your arguments with:		
	*N*	%
High difficulty	1	0.3
Some difficulty	130	32.5
No difficulty	269	67.3
Interpretation:		
	*N*	%
Positive for Criteria 1	107	26.8
Positive for Criteria 2	4	1
Positive for both criteria	15	3.8
Negative	274	68.4

Regarding the type of violence experienced, among the 126 women who tested positive for the WAST, 79.4% suffered psychological violence, 79 (62.7%) experienced economic violence, and 4 (3.2%) encountered sexual violence (Table 4).

**Table 4:** Type of domestic violence between positive and negative to WAST.

Type of violence			WAST scale	
	Negative		Positive	
	N	%	N	%
Psychological	144	36	100	79.4
Economical	88	22	79	62.7
Emotional	20	5	20	15.9
Physical	10	2.5	9	7.1
Sexual	4	1	4	3.2

Regarding the types of violence reported in the WAST, psychological violence was the most frequent, occurring in 144 cases (36.0%), followed by economic violence in 88 cases (22.0%). Among these, 71 cases (44.7%) of psychological violence and 48 cases (30.2%) of economic violence were associated with a lack of bonding on the Evaluation of Affective Bonding and Prenatal Adaptation (EVAP) scale.

Likewise, when comparing adverse psychosomatic outcomes and negative health behaviors, postpartum depression (using the CES-D scale), and alcohol and/or tobacco consumption were higher among the women who experienced violence. Women with postpartum depression had nearly three times the odds of a positive WAST result compared with those without depression (OR = 2.95; 95% CI: 1.87–4.63). Alcohol consumption modestly increased the odds (OR = 1.51; *p* = 0.041), whereas tobacco use more than doubled the likelihood of a positive WAST score (OR = 2.28; *p* = 0.003) (Table 5).

**Table 5:** Psychosomatic outcomes and health risk behaviors as scored on the WAST scale.

Psychosomatic outcomes	WAST scale						OR	IC 95%	*p*-Value
			Positive		Negative				
	N	%	N	%	N	%			
			126	31.5	274	68.5			
Postpartum depression	117	29.3	57	45.2	60	21.9	2.946	1.87–4.63	0.000
Alcohol consumption	241	60.3	87	69	154	56.2	1.509	0.97–2.31	0.041
Tobacco Consumption	145	36.3	61	48.4	84	30.7	2.282	1.31–3.94	0.003
Drug use	4	1%	2	0.5%	2	0.5%	2.194	0.30–15.75	0.375

In the multivariable logistic regression model, postpartum depression was independently associated with a positive WAST result (OR = 2.87; 95% CI: 1.80–4.57; *p* < 0.001). Tobacco consumption also remained significantly associated with higher odds of WAST positivity (OR = 2.20; 95% CI: 1.18–4.11; *p* = 0.014). Alcohol consumption (OR = 0.98; 95% CI: 0.59–1.62; *p* = 0.938) and drug use (OR = 1.32; 95% CI: 0.16–10.60; *p* = 0.795) were not independently associated with WAST outcomes in the adjusted model (Table 6).

**Table 6:** Multivariable logistic regression análisis of factors associated with a positive WAST result.

WAST positive (*n* = 126)	Variable	*n*	%	B	SE	Wald	df	OR	95% CI	*p* value
	Postpartum depression	57	45.2	1.055	0.38	19.664	1	2.871	1.80–4.57	0.000
	Alcohol consumption	87	69	−0.20	0.59	0.006	1	0.980	0.59–1.62	0.938
	Tobacco Consumption	61	48.4	0.787	0.31	6.087	1	2.197	1.176–4.106	0.014
	Drug Use	2	0.5	0.26	1.063	0.68	1	1.318	0.164–10.595	0.795

B, log coeddicient; SE, standard error; OR, odds ratio; Wald, wald test statistic; df, degrees of freedom.

Of the total study population, 109 women (27.3%) suffer from one or two types of violence, even if their responses were less frequent than “sometimes.” Additionally, 4.4% (17 women) report experiencing three to five forms of violence (Table 7).

**Table 7:** Frequency of each type of violence.

Type of violence	Frequently	Sometimes	Never
Psychological	21 (5.3%)	123 (30.8%)	256 (64.0%)
Economical	15 (3.8%)	73 (18.3%)	312 (78%)
Emotional	4 (1%)	16 (4%)	380 (95%)
Physical	3 (0.8%)	7 (1.8%)	390 (97.5%)
Sexual	2 (0.5%)	2 (0.5%)	396 (99%)
One type of violence	68 (17%)		
Two types of violence	41 (10.3%)		
3–5 types of violence	17 (4.4%)		

Regarding adverse perinatal outcomes, the most frequent event was urgent hospital admission (71.8%) which showed statistical significance (*p* = 0.044) when comparing exposed and non-exposed women. Other common outcomes included delivery by cesarean section (86.4%), threatened preterm labor (22.8%), preterm delivery (32.8%), and admission to neonatal intensive care unit (39.8%) (Tables 8, 9).

**Table 8:** Frequency of adverse perinatal outcomes.

Variable	Frecuency	Porcentaje (%)
Reason for hospitalization		
Elective	113	28.3
Urgency	287	71.8
Procedure		
Vaginal delivery	45	11.3
Ceasarean	343	86.4
Abortion	5	1.3
Uterine curettage	4	1.0
Amniotic fluid disorders		
Yes	58	14.5
No	342	85.5
Gestational hypertension		
Yes	23	5.8
No	377	94.3
Preeclampsia		
Yes	18	4.5
No	382	95.5
Obstetric hemorrhage		
Yes	20	5.0
No	380	95.0
ICU stay		
Yes	27	6.8
No	373	93.3
Threatened preterm labor		
Yes	91	22.8
No	309	77.3
Preterm birth		
Yes	131	32.8
No	269	67.3
Threatened abortion		
Yes	75	18.8
No	325	81.3
Miscarriage		
Yes	10	2.5
No	390	97.5
IUGR		
Yes	37	9.3
No	363	90.8
Premature rupture of mebranes		
Yes	49	12.3
No	351	87.8
Admission to NICU of the newborn		
Yes	159	39.8
No	241	60.3

ICU, intensive care unit; IUGR, intrauterine growth restriction; NICU, neonatal intensive care unit.

**Table 9:** Risk of adverse maternal and neonatal outcomes associated with domestic violence exposure in pregnancy.

Variable	Domestic violence		Odds ratio	95% CI		*P* value
	No, *n*	Yes, *n*		Lower	Upper	
Reason for hospitalization						
Elective	85	28	0.726	0.507	1.039	0.044
Urgency	189	98	1.142	0.998	1.307	
Amniotic fluid disorders						
No	240	102	1.387	0.981	1.963	0.057
Yes	34	24	0.835	0.666	1.048	
Gestational hypertension						
No	259	126	1.111	0.623	1.983	0.443
Yes	15	8	0.949	0.699	1.289	
Preeclampsia						
No	262	120	1.061	0.543	2.073	0.523
Yes	12	6	0.972	0.696	1.357	
Obstetric hemorrhage						
No	262	118	1.288	0.738	2.249	0.271
Yes	12	8	0.870	0.605	1.253	
ICU stay						
No	261	112	1.727	1.163	2.564	0.18
Yes	13	14	0.688	0.463	1.023	
Threatened preterm labor						
No	218	91	1.306	0.956	1.784	0.068
Yes	56	35	0.872	0.730	1.042	
Preterm birth						
No	187	82	1.102	0.816	1.489	0.303
Yes	87	44	0.955	0.826	1.105	
Threatened abortion						
No	228	97	1.296	0.931	1.802	0.091
Yes	46	29	0.874	0.721	1.061	
Miscarriage						
No	267	123	0.951	0.365	2.479	0.610
Yes	7	3	1.022	0.678	1.543	
IUGR						
No	246	117	0.755	0.419	1.358	0.214
Yes	28	9	1.117	0.918	1.358	
Premature rupture of membranes						
No	243	108	1.194	0.801	1.780	0.246
Yes	31	18	0.914	0.730	1.144	
Admission to NICU of the newborn						
No	166	75	1.031	0.768	1.383	0.463
Yes	108	51	0.986	0.860	1.130	

CI, confidence interval; ICU, intensive care unit; IUGR, intrauterine growth restriction; NICU, neonatal intensive care unit.

**Figure 1:**
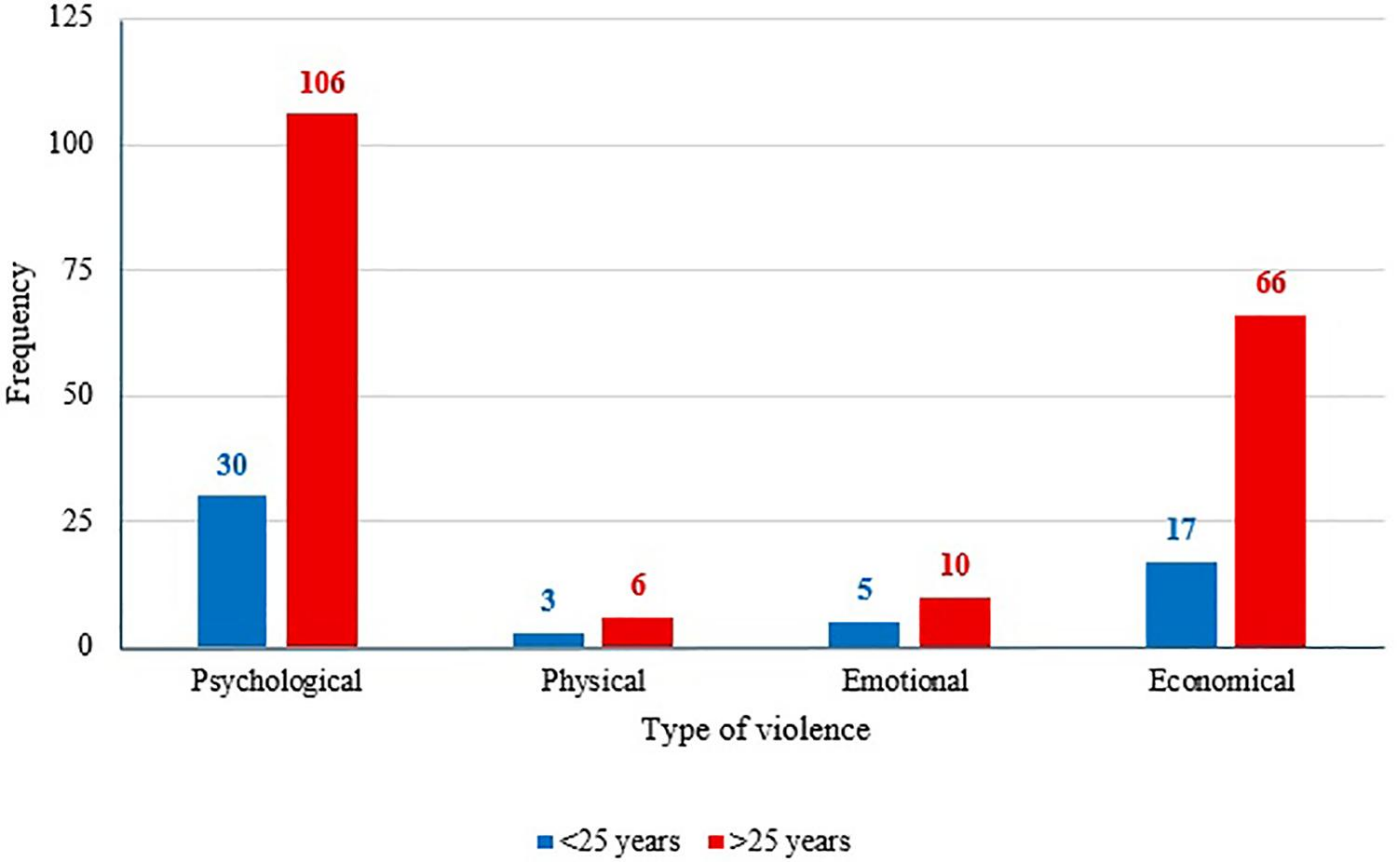
Frequency of type of violence by age groups.

**Figure 2:**
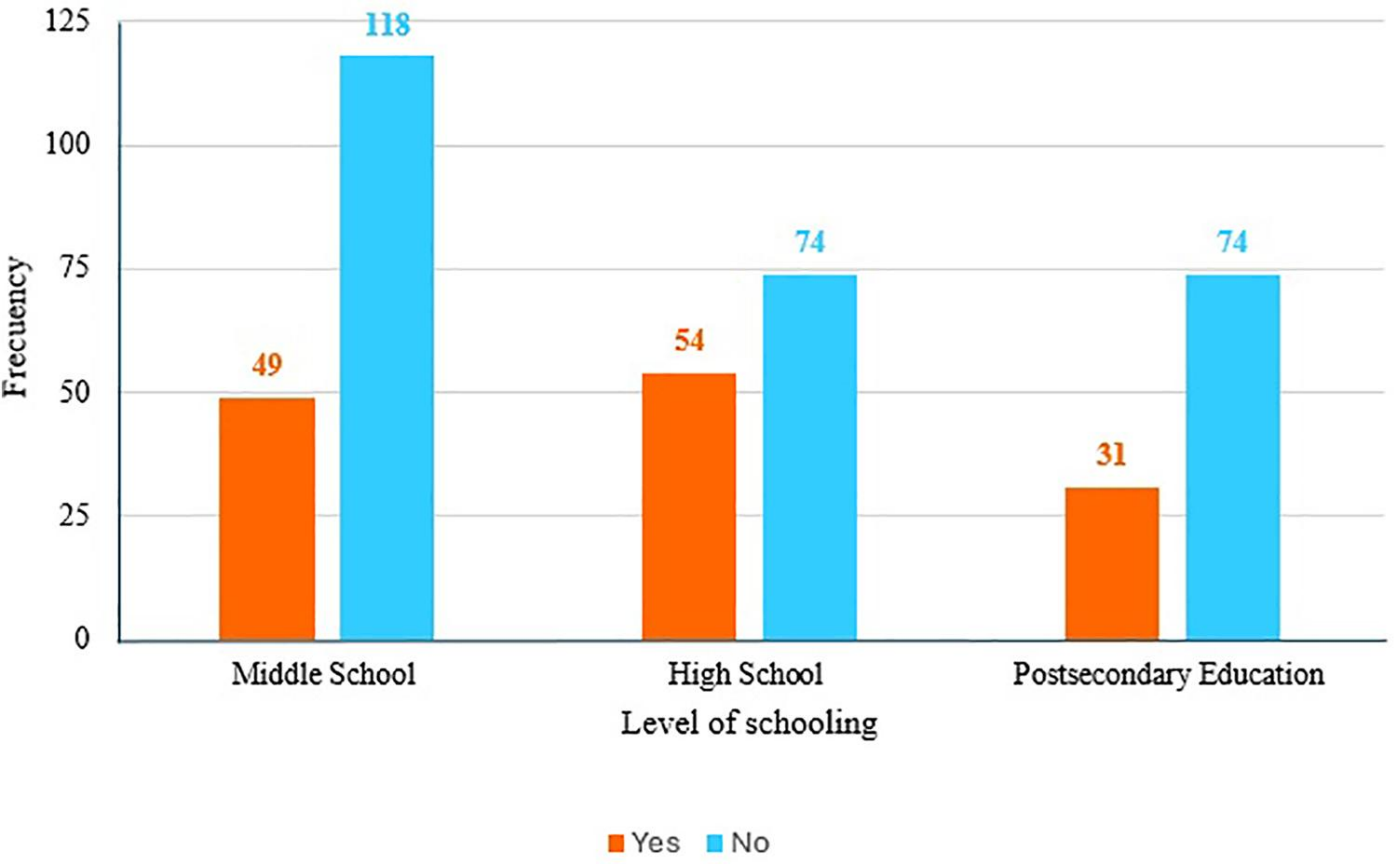
Presence of domestic violence according to level of schooling.

Women aged 25 and older reported more frequent instances of violence, but one-quarter of women under 25 also experienced violence (Figure 1). When comparing domestic violence based on the level of education, we observed that 79% of the victims had basic or secondary schooling (*p* *=* 0.052, Figure 2).

Regarding clinical and demographic characteristics, we found that living with a partner was a risk factor for domestic violence, with 100 out of 126 women (79.4%) affected. Additionally, 24.6% of the victims did not want the pregnancy, and 60.3% of the cases involved unplanned pregnancies. About 30.2% of the women had a history of abortion, and 7.5% (30 women) did not receive prenatal care. Among the 215 women with pre-pregnancy obesity, 77 (61.1%) reported experiencing some type of violence (*p* *=* 0.045).

### Implications

These findings have important public-health implications for clinical care, mental health support, and policy development. The observed prevalence and associated factors highlight the need to strengthen routine screening for domestic violence during pregnancy as part of standard antenatal care. Early identification can facilitate timely referral to psychological support services, improve maternal and fetal well-being, and inform institutional and governmental policies aimed at preventing violence and protecting vulnerable populations.

### Strengths and limitations

The study's main strength lies in the utilization of an internationally validated and Spanish-validated instrument on domestic violence against women, which enabled us to obtain a frequency comparable to that estimated by WHO and INEGI. However, the study population was drawn from a tertiary-level hospital, representing a weakness due to the vulnerability of patients with severe pregnancy complications. Additionally, some women in the non-violent group responded affirmatively to one or more WAST items, suggesting that they may have been experiencing abuse unbeknownst to them. Therefore, the frequency of violence may have been underestimated. Another limitation is its cross-sectional design does not allow for causal inferences, as it captures associations at a single point in time. Additionally, the use of self-reported measures introduces the possibility for sensitive variables. Finally, because the study was conducted at a single institution, the generalizability of the findings to other settings or populations may be limited. Further research in broader and more diverse samples is warranted.

## Discussion

Over the past decade, an estimated 736 million women, representing one in three women, have endured physical or sexual violence from an intimate partner or sexual aggression from others. The incidence of domestic violence observed in our study stands at 31.5%, akin to the 30% cited by the WHO and lower than the 43% reported by INEGI in 2018 (28, 29).

Various studies report on domestic violence and its adverse health outcomes for both the mother and the fetus (11). One such study, conducted by Hawcroft et al., included a systematic review and meta-analysis of 41 articles (29 studies) with a total of 19,101 participants from 10 countries. The studies reported joint estimates of lifetime prevalence of exposure to any type of IPV at 73.3% (64.1–81.6), physical violence at 35.6% (24.4–47.5), sexual violence at 22% (13.3–32.0), and emotional/psychological violence at 49.8% (37.3–62.3). Furthermore, exposure to domestic violence was associated with increased odds of adverse health outcomes: depression (OR: 3.3, 1.7–6.4), sleep problems (OR: 3.2, 1.5–6.8), abortion (OR: 3.5, 1.2–10.2), pain (OR: 2.6, 1.6–4.1), and hypertension (OR: 1.6, 1.2–2.0) (30).

One systematic review and meta-analysis, conducted by White et al., synthesized findings from 201 studies, collectively encompassing data from 250,599 women and research across 46 countries, primarily from high-income countries. The pooled estimates lifetime prevalence of exposure to any type of IPV was found to be 37.3% (95% CI [30.6%, 44.6%], k = 31, I2 = 99.3%), physical violence 18.3% (95% IC [13.5%, 24.4%]), sexual violence 9.6% (95% IC [7.0%, 13.0%]), and psychological violence 32.8% (95% IC [23.1%, 44.0%]). Furthermore, exposure to domestic violence was associated with increased odds of adverse health outcomes: depression (OR: 2.24, 95% CI [1.70, 2.94]), anxiety (OR: 2.34, 95% IC [1.91, 2.77]), psychological distress (OR: 3.42 [95% CI 2.80, 4.18]), suicidal ideation (OR: 3.14, 95% IC [2.70, 3.66]) (31).

Likewise, Martínez-Galiano et al. (32) found that among 141 women interviewed during pregnancy, 31.2% (44 women) reported being victims of partner violence. Factors predisposing to partner violence during pregnancy included a low level of education, partner unemployment, living with dependents, being single, multiparity, lack of stable employment, and having an unwanted pregnancy (p ˂ 0.05).

In 2021, Mella et al. (33) reported a prevalence of violence against pregnant and postpartum women of 5.7% and 5.9%, respectively. They identified associated factors such as being an immigrant, having a history of domestic violence, not having a supportive partner, and partner alcohol consumption.

These findings were like those of Hawcroft et al. (26), who reported that emotional/psychological violence was the most frequent type at 49.8% (37.3–62.3), followed by physical violence at 35.6% (24.4–47.5). Wassie et al., found that of 701 women interviewed during pregnancy, 34.8% were victims of at least one form of domestic violence, with emotional violence being the most common at 23.8% (34).

Additionally, we found that most patients who suffer from domestic violence experience a lack of emotional bonding and prenatal adaptation. This may be due to the fear of assuming the responsibility of caring for themselves and the newborn, or because the unplanned pregnancy may be the result of sexual violence. These findings are consistent with those reported by Ma and Zhang in 2025. In their systematic review and meta-analysis of 37 observational studies involving 36,214 pregnant women, the authors found that women with undesired pregnancies had significantly higher odds of experiencing IPV compared with those whose pregnancies were planned (OR = 2.31, 95% CI: 1.13–4.73) (35).

Regarding adverse perinatal outcomes, preterm delivery, admission to neonatal intensive care, and low weight for gestational age were the most common outcomes found in our study. We also observed that most of the patients who had been raped had lower educational levels compared to those who had not been raped. Basic and middle schooling were most frequent among patients suffering from domestic violence. This could be because uneducated pregnant women may lack the ability to communicate effectively with their partners to resolve disagreements. Evidence shows that low levels of education and lack of decision-making power increase the likelihood of women experiencing domestic violence during pregnancy (36).

This observation is consistent with previous studies, including a systematic review and meta-analysis by Bifftu and Guracho, which evaluated 13.912 women and reported a domestic violence prevalence of 37%. In their analysis, several pregnancy-related factors were associated with IPV: low educational level (OR = 3.88; 95% CI: 1.48, 6.27), unplanned pregnancy (OR = 1.77, CI: 1.48, 2.05), and late initiation of prenatal care (OR = 0.30; 95% CI: 1.15, 1.44) (37).

Collectively, these results highlight the significant contribution of educational, reproductive, and healthcare-seeking factors to women's risk of experiencing domestic violence during pregnancy. In particular, the recurrent association between unintended pregnancy and elevated rates of IPV underscores the increased vulnerability of women confronting an unplanned gestation, emphasizing the importance of tailored screening and support strategies for this high-risk population.

## Conclusion

Our findings are in line with those of other studies, confirming the pervasiveness and gravity of domestic violence. It is concerning that around one-third of the women in our study experienced IPV during pregnancy. Psychological and economic abuse were the most prevalent forms of mistreatment. These women also grappled with issues such as unwanted pregnancies, difficulties in forming bonds and adjusting to pregnancy, postpartum depression, increased tobacco use, and lower levels of education.

## Data Availability

The original contributions presented in the study are included in the article/Supplementary Material, further inquiries can be directed to the corresponding author.
